# A Systematic Narrative Review of Implementation, Processes, and Outcomes of Human Library

**DOI:** 10.3390/ijerph20032485

**Published:** 2023-01-30

**Authors:** Gary Yu Hin Lam, Hei Ting Wong, Mengge Zhang

**Affiliations:** 1 Department of Educational Psychology, The Chinese University of Hong Kong, Shatin NT, Hong Kong, China; 2 Cultural Studies in Asia Programme, Department of Communications and New Media, National University of Singapore, Singapore 117416, Singapore

**Keywords:** human library, living library, diversity, stigma, community-based intervention

## Abstract

First started in Denmark in 2000, Human Library (HL) has been adopted by different communities around the world. It is an innovative approach that engages “readers” from the general public to have collaborative conversations with “books” from minority or marginalized communities to learn about their lived experiences and reduce public stigma and stereotypes. While the HL is popularized, its research base and implementation structure remain limited. This systematic narrative review aims to review the HL literature to (1) summarize the design, implementation, processes, and outcomes of existing HL programs and (2) synthesize recommendations for future implementation of more effective, ethical, and sensible HL. A systematic search in eight electronic databases yielded 23 journal articles and book chapters about HL published from 2010 to 2022. The identified literature demonstrated variations in format, venue, scale, preparation, and recruitment. A wide range of books with different social identities and from different cultural groups were reported, while readers were mostly from university and school communities. Reduced prejudices and improved attitudes were reported in readers, while both readers and books reported various facets of personal growth. Future HL using implementation guidelines with clearly articulate ethical considerations and diverse rigorous research methodologies are recommended.

## 1. Introduction

Human Library (HL), also called Living Library previously, is similar to a traditional library where “readers” can read “human books” who share their life experiences and stories about topics that are prejudiced or misunderstood in society. The first HL, organized in response to the murder of the founders’ mutual friend in Denmark in 2000, had an explicit goal to educate young people to prevent violence in the community [1]. Since then, HL’s target groups have extended beyond youth and the embedded messages have extended beyond anti-violence. HL gained much traction across the globe as a method to bring together people of diverse backgrounds for conversations to learn about each other. In its contemporary rendition, HL has expanded to “better our understanding of diversity” and “to challenge prejudice, get aim to help rid discrimination, prevent conflicts and contribute to greater human cohesion across social, religious and ethnic divisions” [2]. According to the official website of the registered Human Library Organisation, HL has been held globally in over 80 countries, featuring numerous types of human books and readers [2].

These goals to eradicate public stigma and promote community inclusion are ambitious and aspirational. There are surprisingly few large-scale contact-based interventions developed to promote intergroup harmony, as they are difficult to be implemented and tested, especially in naturalistic community settings [3]. In the case of HL, compared to its widespread adoption across different communities and regions globally, research literature on HL is disproportionately scarce, and its evidence base for program effectiveness is extremely slim. The interests in studying HL systematically and intellectually can be reflected in the growing body of “gray literature” (e.g., magazines, monographs, conference papers) that described the development and evaluation of numerous HLs (e.g., [4]). Although this body of literature can provide anecdotal information that may benefit discussion and promotion of innovative and emerging ideas [5], they lack rigorous research designs and systematic reporting standards that are necessary to build a strong and comprehensive evidence base. In the long run, more high-quality research is needed to support sustainable uptake and dissemination of HL initiatives that can live up to the promise of HL in the real world. Not only does HL lack systematic documentation and rigorous empirical evidence, its conceptual analysis and process of change are also underexplored. Numerous questions remain unaddressed: How is the process of HL that can induce changes in readers? What preparation work is necessary to facilitate the storytelling and interaction in HL? What ethical considerations are necessary to protect both books and readers from emotional and psychological harm? How can HL balance its educational goals with precautions so that participants can benefit from new learning but not overly emotionally challenged?

In response to these unaddressed questions and pertinent concerns, this article aims to review the current body of knowledge on HL by conducting a systematic narrative review on the existing literature. Specifically, we aim to summarize past implementation and description of HL in the research literature in order to (1) understand the focus of HL in terms of its intended objectives and target participants (i.e., readers and books), (2) investigate how the HL is organized and how its process is facilitated, and (3) review the outcomes and effectiveness of HL. Through examining the empirical descriptions and findings of HL, we also critically reflect on the ethical issues that are implicated in the design and process of HL to generate recommendations for future practice and research of effective, ethical, and sensible HL.

## 2. Method

A systematic narrative synthesis approach was used to review the academic literature on HL following the established guidelines from the Economic and Social Research Council in the United Kingdom [6]. Due to the large amount of exploratory and descriptive studies that are qualitative in nature within the existing body of HL literature, a quantitative synthesis (e.g., meta-analysis) is not possible or appropriate. Instead, a systematic narrative synthesis has its strength in summarizing both quantitative and qualitative studies which are relatively small in samples, while retaining the rigor of using systematic and explicit methods to review all relevant evidence [6,7]. The search procedures were conducted in accordance with the PRISMA guidelines [7,8]. Research literatures were identified by searching eight electronic databases including APA PsycINFO, APA PsycBOOK, Medline, ERIC, CINAHL, PubMed, Web of Science, and ProQuest Social Science Database. Search terms “human library” or “living library” were used to identify keywords anywhere in the full text of publication. Intellectual works included in this review have the following elements: (1) The primary focus of the publication, or at least part of it, is on HL or any of its components; (2) Published as peer-reviewed journal articles or book chapters. Due to the varied quality in the gray literature, commentaries, magazine entries, and conference papers were excluded.

**Figure 1:**
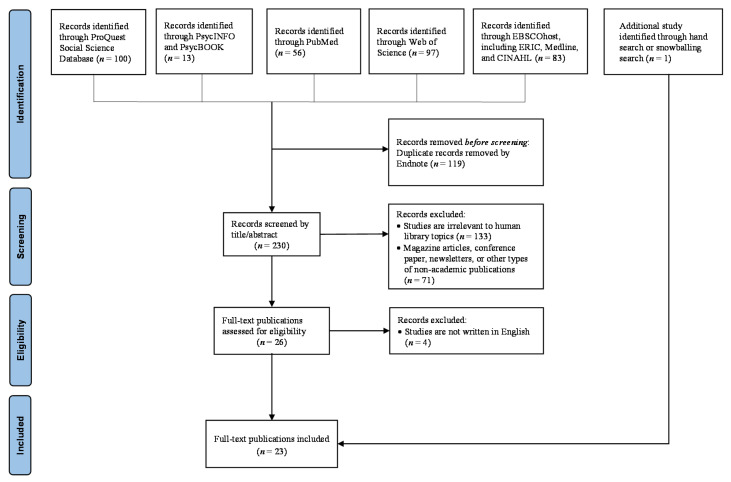
PRISMA diagram of search process and results.

Figure 1 illustrates the search process and the overview of search results. Initial search yielded 230 unduplicated results dated from the 1800s to September 2022. All authors reviewed the titles and abstracts to determine topical fit based on the above inclusion criteria. Majority of publications were excluded due to topical mismatch (i.e., referring HL to the collection of human genome or library system management/development, focusing on oral history). Two articles written in Spanish [9,10] were excluded due to limited resources to ensure the quality of translation completed by automated translation engine. The decision to not use any research quality appraisal tool was to maximize the conceptual contribution of the broad array of existing literature to understand how HL is supposed to be instead of its actual research or implementation quality [11]. The final sample in this review consisted of 23 publications (21 journal articles and 2 book chapters) [12,13,14,15,16,17,18,19,20,21,22,23,24,25,26,27,28,29,30,31,32,33,34].

The first author read the full text of all the publications and extracted the following information using a Microsoft Excel spreadsheet: (1) article information (e.g., published date, location, research design, measurements); (2) goals of HL and research objectives; (3) characteristics of human books and readers (e.g., number, demographic information, social group backgrounds, recruitment, selection); (4) how HL is implemented (e.g., organizers, location, time, facilitators); (5) effects of HL; (6) other important issues related to HL. To establish reliability, 22% (five publications) of the sample were reviewed and extracted by both first and third authors. Data were largely in agreement with each other, while minor discrepancies were discussed to resolve disagreement and reach final consensus. Results were summarized below to analyze how HLs were designed and implemented, and how research was conducted to inform the evidence base for HL. Characteristics of the included studies were also summarized in Table 1.

## 3. Results

### 3.1. Publication Characteristics, Study Objectives, and Research Designs

**Figure 2:**
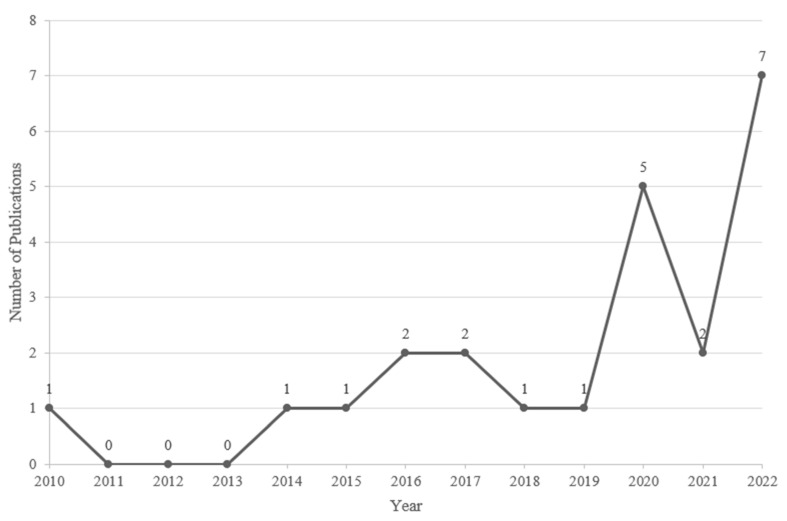
Number of HL publications included in the review across years.

Interest in studying HL increased exponentially in the past decade (Figure 2). HL events reported were located across different continents, including North America (*n* = 8: The United States (USA), Canada), Europe (*n* = 6: The United Kingdom (UK), The Netherlands, Hungary, Poland), Asia (*n* = 6: Hong Kong, India, The Philippines, Kazakhstan, China), Oceania (*n* = 1: Australia), and other (*n* = 1: Turkey, which is part Europe part Asia). HL was researched in incredibly diverse countries, although most studies came from the US (*n* = 4), Canada (*n* = 4), and the UK (*n* = 3).

Fourteen (61%) publications were descriptive in nature [12,13,14,16,17,19,22,25,26,27,30,31,32,34], most of which aimed to “describe”, “overview”, “review”, “reflect on”, or “discover” the HL approach and reported on the implementation, process, and experience of HL events. Many of their research methodologies are of questionable quality, as they did not describe the exact research designs, mentioned the use of case study without details, or used generic methods to collect the outcomes or observations after an HL (e.g., only post-event survey, observation, or interview). Five articles (22%) [15,20,21,24,28] used pre-/post-evaluation to compare participants before and after HL, two of which included a control group [15,21], and another one used random assignment in an experimental setting [28]. Others utilized systematic qualitative designs, including interview study, phenomenological case study, practitioner inquiry group, and content analysis [18,23,29,34]. All publications were focused directly on the HL events, except that two studies respectively reported on the development of a data system for managing HL resources [31] and a content analysis of documents about HL standards [18].

### 3.2. Organization, Design, and Implementation of HL

#### 3.2.1. Venue and Mode

The reported HLs were held in enclosed spaces. The majority of HLs were reportedly held in physical libraries, such as those associated with universities [14,16,17,19,22,24,25,34] and public libraries [14,17,20,32]. Other venues were primarily in educational settings, including university venues or facilities, student or information centers, and classrooms [17,21,24,28,34]. One article explicitly explained the reason for choosing the venue was to “recognize the university as part of the place where people live and grow up, and therefore part of their identity” [16]. One HL was held in an art gallery in the community in conjunction with an exhibition [13]. Some of the HLs were organized as a part of some naturalistic contexts, such as library initiatives, university programs, diversity week, and collaboration with mental health agencies. In seven publications, strategies to advertise the HL were noted, such as invitation emails, websites, posters, and word of mouth [14,19,21,22,24,30,34]. All of the reported HL events were conducted in-person, except that three HLs were partially or completely online, some due to the COVID-19 pandemic [30,32,34]. Another HL included an online component to appeal to a broader audience and to create a platform for updating and organizing human books [34].

#### 3.2.2. Goals of HL

Across all publications, HL was overwhelmingly described as an approach that can promote humanities and virtues. Its goals ranged from “connecting people”, “experiencing diversity”, “encouraging understanding”, to “hearing different stories”, “learning about life experiences”, “facilitating dialogues”, and ultimately “promoting open-mindedness”, “challenging binary thinking”, and “reducing prejudices and stereotypes”. In four publications, HL was established with explicit educational purposes, such as connecting to specific curriculum of university courses [16,30], fostering career insight into less-known professions [16,26], and promoting internationalization in a university [22]. While HL was understood to be a “leisure-based initiative” [33] and distinct from “formal learning contexts” [27], it was also not meant to be purely fun, entertaining, and “emphatically not intended as an encounter with an exotic figure” [27].

### 3.3. Human Books: Backgrounds, Recruitment, and Preparation

Regarding the human books in HL, an incredible diversity of backgrounds and identities was noted. They included race/ethnicity/nationality (e.g., Roma, Native American, Jew), sexual identity/orientation (e.g., LGBTQ, transgender), religion (e.g., atheist, Muslim, Hindu), (dis)ability/neurodiversity (e.g., mental illness, alcoholism, abuse history, learning difficulties, physical disabilities, autism, HIV+), artistic/cultural groups (e.g., burlesque dancers, graffiti artist, drag queen, tattooed, minimalist), occupations/professionals (e.g., occupational therapist, psychologist, professor, veteran, farmer, web celebrity), other socially marginalized groups (e.g., anti-poverty representative, sex-trade worker, homeless, freegan, inner-city youth, teenage mother, asylum seeker), and many more with intersectional identities (e.g., gay female rabbi, blind social worker). Only three publications documented the books’ demographic information (e.g., age, gender) beyond their presented identities [13,15,33].

The human books were usually acquired within the organizers’ network and connections. The recruitment of books was generally completed by reaching out to the organizers’ personal, social, and professional networks [16,17,21,30], inviting specific individuals or groups based on recommendation or specific themes [19,22,30], and collaboration or consultation with community organizations who had access to specific target groups [13,16,21,22]. Organizers also encouraged members in the community or university to apply voluntarily or nominate others through, for example, an online form or email [14,19,30]. One article described going “door-to-door” to personally invite staff and faculty to become books [30]. Another article mentioned an individual contacted the organizer volunteering to be a book [19]. Intentional involvement with the broader community outside of the expert or immediate network of the organizers was noted across a few articles: The book recruitment was meant to avoid experts [14] or own staff members [30], and there was a gradual shift in their book selections “from experts, scholars and professors within campus to more socially experienced participants” [34]. Only one publication discussed the rationale of not paying the books as “participation is a gift from which both Living Books and Readers derive benefit”, although transport costs were provided [16].

Consistent with the goals of HL, the books generally had unique, challenging, adverse, or unhappy life experiences to show [13,14,19,25]. Specifically, books selected should have “credible narratives” [19] and represent certain perceived stigmatized or marginalized groups in society [21,25] who “may have been ridiculed and discriminated against because of who they are and what they do” [25]. One article noted the importance to consider “culturally relevant social norms, taking into account both globally and locally stigmatized group members in the context” to ensure that book selections were relevant to the local understanding of privilege and oppression [21]. Despite often negative experiences, some books were noted to highlight “resilience”, “pride”, “turning point”, and “passion” [13,14,17,32]. For ongoing or multiyear HLs, book collections were emphasized to be dynamic and diverse to represent varieties of human experiences [19,21,34].

To prepare the books for HL, many articles mentioned the use of titles and/or short descriptions [14,16,17,19,21,24,26,34] to help attract readers and orient them to the focus of the sharing [19,21,26]. Titles could range from a brief label representing the book’s primary identity [21] to more elaborated and figurative versions [14,16,24]. Specifically, while some titles may directly narrate their stories, others may be more subtle, and some books “play with negatives stereotypes and labeling they had encountered” and “question around the differential use of language, and the experiential basis for it, can start to be explored and unpicked” [16]. The use of visual book cover was also evident [26]. Books might write the descriptions themselves or accomplish that by receiving assistance from the organizers [33]. In the analysis of HL standards, a role of the organizers was to “cooperatively develop descriptions” with the books and ensure their descriptions include stereotypes and prejudices, despite the book as the primary source of authority [18]. Another article [21] reported that a book was not satisfied with the organizer’s polishing the title for him/her (i.e., “ex-prisoner” instead of “ex-political prisoner”). In two publications, books were required to wear an official T-shirt [12,24]. In one of the HL events, the T-shirt was intended to have “their diagnosis written on the back, illustrating how the general public often use diagnoses as a label and do not see the individual behind it.” [12].

### 3.4. Readers, Reading Preparation, and HL Process

Regarding the readers, 11 publications (48%) reported students were their primary audience, either in universities [14,16,17,19,21,22,24,26,30,34] or high schools [15,17]. Some included the involvement of other members in the campus or community at large, including faculty, staff, and residents [14,17,19,21,22,24,25,31]. Several HLs with specific educational objectives were dedicated to targeted students of certain majors, courses, or demographics [16,17,22,26,34]. Only three publications (13%) described HLs were intended for the public [12,13,20]. Based on the available demographic information, these readers were majority female, Caucasian, completed higher education, and in young or mid adulthood [13,20]. Some instances where human books became future readers, and vice versa, were noted [16,19].

Advance preparation in terms of training, orientation, or briefing was completed in a range of weeks in advance, days prior, or right before the HL event. Nine publications (39%) referred to some kinds of rules, guidelines, or expectations to be explained to the participating books and readers [15,16,19,20,21,24,28,32,33], so everyone was aware of what to expect [16,24]. Common reminders included mutual respect [21,24,28,32] and confidentiality [21,25]. Books were reminded to be genuine and truthful in their sharing [21,24,28], while readers should “accept the rights of the book” [32] and “return the book in the same mental and physical state as they received it” [21,32]. While some publications emphasized the full control of the books over their own story (e.g., whether to bring up or answer certain questions, and how much time they want to spend in a conversation or event [16,19]), others appeared to reiterate the rights on both the books and the readers [21,32]. Only one article included a supplementary document with a comprehensive list of reminders for books and readers [21]. Apart from didactic presentation of rules and expectations, other training strategies highlighted in the publications included modeling by the organizers [16], role-play simulation among the books [34], discussion about ways to respond to inappropriate or uncomfortable questions [34], conversation to clarify stereotyping terminology [15], and review case examples [15].

The actual HL events reportedly lasted from one to a few hours, occasionally a full-day event. Each reading session typically lasted around 15 to 20 min, with some longer sessions (30 to 60 min) evident [12,16]. In some publications, one reader was paired with one book per session [13,22]. One article explicitly stated that “two readers can borrow the same book if they know each other and if the book agrees” [32]. In others, small groups of 3 to 10 readers were reported. A large group of 25 students in a classroom setting [24] and 200 attendees in a lecture-style event were also reported [34]. Consequently, books were visited and read once or multiple times depending on the structure of the HL, although some books were reported to have relatively low check-out rate [24] or even no visit [14]. Four publications mentioned the availability of refreshment during the event [16,19,21,22].

Common physical space to place books were separate rooms or classrooms [19,21,24]. It was important to have “some degree of privacy for conversations” [19], “sit freely and feel relaxed”, and “a safe space for communication with minimal pressure” [28]. High-traffic areas such as the main corridor of the university library could be chosen to facilitate movement and increase publicity [17,22].

The check-out process varied largely across publications. One HL indicated that readers can only focus on one book [24], while others allowed readers to read multiple books [20,28]. Some HLs emphasized that books cannot be reserved [32], and readers were free to browse and choose the books they wanted to read by going around the venue [24]. In others, reservation of specific time slots using a “library card” was required to facilitate book selection and scheduling [21,26]. Readers in another HL were instructed to select a particular group and then matched with an assigned book [15].

The interaction during a HL reading session was described to be like “student-led small group teaching” [16], “book-club meeting with the author” [16], or conducting an “interview” [34]. Although some conceptualized the HL session as “not a presentation”, “no PowerPoint”, “no handouts”, can bring “point-form notes”, and an “informal conversation” [19,24], formats such as presentation, teaching, lecturing, and performance were incorporated in some HLs [30,34]. Books and readers were provided with guiding questions, generic conversation starters, and simple prompts to facilitate the general directions of the conversation [17,21,30]. The conversation often led by the books; it either began with the books telling their story [28,34] or them speaking for most of the time [19]. Readers then asked questions, gave responses, shared their experience reading the book, and discussed further the specified issues [15,16,19,28,33]. In some HLs, readers could ask books whatever questions they wanted [15] and books were “totally open” and “happy to answer” any questions [27]. More importantly and connected to the aims of having a HL, the conversation was intended to be focused on the identified prejudice or specific topic of the event, such as acculturation and rehabilitation experiences [15,22,24,28]. A closing plenary or an overall question-and-answer session were sometimes used where participants can exchange questions and reflect on their learning [16,30]. In some HLs, readers were asked to draft questions for the books in advance [15], write mandatory reflection [16], and write feedback and messages of appreciation for the books [30]. While some publications mentioned providing readers with book titles and descriptions for selection and getting familiarized with the topic [17,26,33], it was implied in other articles that readers would have received adequate knowledge about the books’ backgrounds. One publication mentioned the readers were not tracked by their demographics and would disclose their personal information voluntarily in the conversations with books, which were concerned by some of the books [22].

Besides the books and readers, there are supporting personnel, usually served by the hosts or volunteers of the events, involved in running the events and/or the HLs. There were “librarians” assisting the facilitation of HLs. They helped readers to navigate the catalog of books [17], provide directions to books and reading areas [21,22], ensure and maintain the dialogs in a respectful and safe manner [16,17,22], keep time [17,22,33], and stay close by to offer support and answer questions if needed [17,22,33]. Support staff members were there to manage the check-out counter or schedule [17,32]. Translators, also called “dictionaries” in a HL, were evident in some HLs [20,32]. Greeters explained the HL process to participants [17]. Only one publication emphasized deliberately not to “vet” the books during the event because “the decision on whether to participate is left to a Living Book to take, sometimes in discussion between them and their supporters” [16].

### 3.5. Outcomes and Effectiveness of HL

Overall, participants perceived HL as “a space to push themselves” [13] and “bring different social and ethnic groups together” [32], for both books and readers to engage in open communication [17] and listen to stories [25]. This space was dedicated to adult and perhaps public educational purposes [13], and intentional for having dialogues with “people they do not talk to in their everyday life” [32] and about things that “are ordinarily kept silent” [14] or rarely talked about [13,27]. It was commonly reported that readers and books come to HL feeling nervous [13] and with “the baggage of the narrative” [29]. Many readers were also curious, intrigued, and entertained [13,17]. As participants overcame the strange, embarrassing, and uncomfortable feelings over the course of the HL [13,14,17,32], they ultimately appreciated HL as a challenging yet satisfying and eye-opening experience [17,26,30,32].

To readers specifically, firstly, they were not only able to access the books’ direct, personal narratives of experiences, struggles, and worldview, but also communication of their courage, eagerness, yet calmness during their sharing [16,19,25]. Second, readers reported learning something new, meaningful, and inspirational [17,30]. They appreciated the diversity of topics in HL [24] and gained different perspectives and varied answers on different topics and questions [14,22]. For example, readers became more aware of the different dimensions and intersectional identities of a person [13] and the different trajectories one can take in life [17]. Third, readers engaged in “intentional debate” and “critical dialogue” with the books, the contents of which may include difficult social topics (e.g., gender power and diversity) rarely discussed outside of HL and unsafe ideas that discomfort may be induced. Ideally, such “cultural shocks” would lead participants to question binary thinking, societal norms, and power issues [13,17,27]. The conversation within HL being highly personally connected helped the readers to be open to learning about what had been misunderstood and controversial, to “see others as human”, and experience “personal growth” [13,27]. Fourth, readers found the HL experience “mind-blowing”, “eye-opening”, “astonished”, and “surprised” [13,17,19]. They appreciated the openness, willingness to share, and lack of judgment in the conversations with the books, who were strangers to each other [13,17,32]. The HL was also a reflective experience to the readers as they “connected on a deeply personal level with books” [17] and learned to reflect on their own experiences, personal qualities, and responsibilities that may impact others in society [13,17,32].

Assessing the effects of HL on readers was the primary focus of many publications, the majority of which investigated changes in readers’ attitudes. Several publications narratively reported in eliminating prejudices, stereotypes, and fear, as well as enhancing positive attitudes and empathy towards diversity and minority groups [19,23,24,26,32] that went “beyond an intellectual understanding” [23]. Other studies used pre-/post-survey and experimental methods to directly evaluate the effectiveness of HL. HL was effective in enhancing readers’ attitudes in terms of reduced social distance towards targeted social groups [15,20,28], reduced prejudiced attitudes (e.g., racism [15] and mental health stigma [28]), and enhanced positive feelings towards working with people from diverse backgrounds in the workplace [20]. Increased empathy was found in a series of studies targeting different social groups in a publication [21], although another study reported no significant change in empathy-related outcomes [32]. Trust was also reported to be difficult to change [21].

Regarding knowledge, readers across various publications expressed gaining new knowledge about the situation of the books and minority groups [32] and about society, culture, and different beliefs in general [17], as well as receiving answers to their own questions and doubts [14]. Despite the increase in self-perceived knowledge [21], knowledge tests about specific social groups found no change in actual knowledge [28]. Actual behaviors and long-lasting actions were less reported, possibly due to the non-experimental set up of the research on HLs. One should be aware of the possible difficulties in conducting longitudinal research on attitudinal changes. Behavioral intention (e.g., willingness to talk to, develop friendship with, and support collective action for outgroup or minority) was the most difficult to change among other attitudinal factors [21]. A few publications reported possible enduring effects of HL in connection with professional learning and practicing [16,26]. Participants would continue discussion outside of the HL event [17], attend the HL again [17,24], and recommend others to join as books or readers [24,26,32].

For the experience of books, firstly, HL offered them the opportunity to speak to people and raise awareness on topics less known to others [14,29]. Many expressed the desire to share their stories, counter misconceptions, shift the discourse, and influence others’ practices [16,33]. At the same time, books’ sharing went beyond “from monologue to dialogue.” The books realized they learned from others as much as they shared, in which they found similarities and connection with readers and shared purpose and collegiality with other books [17,29]. They also reflected and questioned themselves [14], while showing “a deeper appreciation of the relationship between the I and the not-I by engaging in a critical consideration of the way that humans share commonality and difference” [27]. Thirdly, through the interaction in HLs, the books experienced personal growth and developed insights about their personhood and identities, which also led to their self-understanding and self-acceptance [23,33]. They found it meaningful to share their stories and struggles as they were well-received and valued by others [14,29,33], and they could “focus on redemptory power rather than residual pain” [17]. Lastly, books were found to shift from initial fear of being vulnerable to feeling “liberated”, “gratifying and humbling” in sharing their authentic self through the open and honest conversations [17,25,33]. The process of narrating and (re)presenting themselves indeed helped the books to experience catharsis, move beyond, find meanings, and “represent themselves without an intermediary” [23,29].

At a community and broader societal level, HL was described to promote awareness and intergroup contact among different social groups and issues (e.g., mental illness). It was utilized to facilitate community engagement and collaboration, enhance utilization of and movement across public or otherwise more restrictive spaces (e.g., university campus), promote diversity, cultivate ideas for collective actions, achieve educational purposes yet expand the traditional notion of education, and, even, raise the profile through media coverage [12,13,14,16,17,19,22,23,30].

Based on the available literature, some potential effective HL practices were identified. First, HL was demonstrated to be more effective than didactic teaching in promoting attitudinal changes [28]. The effects were also likely due to contact with specific social groups rather than mere exposure to any types of diversity [21]. Groups with a long history of deep-rooted prejudices in society appeared to be less susceptible to intervention effects of HL [20]. Inconsistent findings were found between the number of books read and the magnitude of attitude changes [20]. HL may be more effective on readers who hold more prejudicial attitudes to begin with [20]. Having a quality, articulate book and an appealing topic appeared to be more important than identifying books with complex backgrounds or representative of the particular social group [13,14,32]. Having an alternative source of information (e.g., scholarly articles in a course-related HL) may offer a more holistic representation. Based on participants’ suggestions, HL with more extensive and meaningful engagement would be welcomed. For example, offering more time for conversation, offering a greater diversity of books, giving greater freedom in book selection, increasing readers for books with low visit rates, preparing readers’ backgrounds for books to tailor dialogue, allowing longer orientation to facilitate introduction among readers and books, and holding debriefing sessions for the books [14,17,22,23,24].

## 4. Discussion

This systematic narrative review synthesized the existing literature on the design and implementation of HL and its outcomes and effectiveness. Based on the current trends and patterns of HL reported in the literature, we identify several important themes that warrant special attention and further discussions to better promote the future development of HL for the betterment of humanity.

First, despite the rapid uptake and popularity of HL across different communities globally, its research base is comparatively limited. The existing body of literature consists of majority case studies or anecdotal descriptions of HL that lack scientific rigor. Furthermore, we observed inconsistent interpretations of HL concepts and rationales across studies. For example, different formats of interactions or conversations were evident. Additional activities, such as didactic presentations and performances, were observed to complement or entirely substitute the conversations between a book and readers, which were arguably different from typical HL in its nature and perhaps effectiveness. While some argued for the needs of a face-to-face interaction, others recommended a virtual or online implementation, or even asynchronous mode that utilizes a library archive. Only can research that is carefully designed and conducted with systematic data collection (quantitative, qualitative, mixed, or others) produce scientifically acceptable findings that help developers make decisions about future HL programs. It is important for the research design to go beyond the appeals of the intended goals demonstrated in actual programs, which includes the active ingredients, process of change, and theoretical frameworks.

Second, while the aspiration of HL is to eliminate prejudices and promote cultural diversity in the community, most of the HL events reviewed were either dedicated to a particular audience or conducted in relatively controlled and contrived settings, such as university and school campuses. Only few studies reported HLs conducted in public spaces or targeted to the general public. This problem resembled the classic dilemmas of intergroup contact research, as those who are more prejudiced are not interested in intergroup contact, creating biases in the study design, participant selection, and generalization to uninvolved groups [35]. Empirical evidence also suggested more prejudiced individuals are likely to experience more remarkable attitude changes than more acceptable people in contact-based interventions [20,36]. We believe that the attempts to reach out and bring new perspectives to laymen are the core mission of HL. On one hand, designing, advertising, and promoting for HL events need to be tailored to appeal to audience of diverse backgrounds. On the other hand, open recruitment of readers may yield different levels of readiness, openness, and acceptance of diversity. While readers’ participation is voluntary and relatively informal, more structured guidance and preparation can help consolidate their learning through preparatory meetings, debriefing sessions, and the sharing of resources to continue their engagement with specific social groups.

Third, if HL is ultimately applied to the general public, several ethical considerations warrant further scrutiny and research. We are concerned about how much preparation or ground rules are necessary to safeguard both readers and books in their participation in HL while generating a meaningful dialog. The existing HL guideline [1] published by the Council of Europe appears to have a low rate of adoption or mention in the published HL studies. So, surprisingly, it is unclear whether past HL events were organized in accordance with the recommendations, and how many measures were based on the organizers’ own judgments. Even in studies referencing this guideline or adopting similar ethical principles, those HLs were mostly for dedicated readers and in semi-controlled settings (i.e., not completely open or public), where unpleasant events such as heated discussions, censored topics, and offensive comments were sparingly noted. Special attention should be given to minors, regardless of their roles in the HLs. In addition to requesting parental consent for participation [32], the organizers may do more on preparations. For example, select sharing topics appropriate for and relating to youngsters while addressing prejudices, deploy strategies to engage the youngsters in participation, alongside to reduce fear of their sensitive questions or unpleasant comments and keep them engaging in the conversation [24]. The organizers may deploy expectation management strategies, such as equipping the books with interpersonal skills on handling difficult questions, classroom or small-group management skills to facilitate conversation with minors, and advising the readers on how to ask questions in a respectful way. Further investigation is needed to evaluate whether the guideline provides appropriate level of safeguards and how its delivery can be adapted to easily understandable language to engage general lay people in having honest, challenging, yet respectful and comfortable conversations during HL.

Moreover, this European-centric guideline is arguably insufficient and insensitive to diversity issues across different cultures that may impact HL considerations locally. For example, certain tabooed or sanctioned topics that have actual ramifications in different parts of the world, such as women and sexual minority issues in patriarchal societies, political dissidents with views different from the laws, and religion freedom in societies with religious restrictions, to name but a few, could place a limit on the possibility of dialogs and discussions. How HL responds to the local needs in the community while avoiding breaching the laws or acceptable standards is a sensitive issue, which needs to be thoroughly thought through and investigated to advance HL practices and its contribution to societal transformation. Any guidelines need to be ethically sound while flexible enough to accommodate needs among the books, readers, and the related communities of different cultures and customs.

Related to this concern, when facing an audience of the general public, books may find it more intimidating to come out and disclose one’s past experiences and challenges. The storytelling experience and the expressed narratives of the books may be very different when speaking to a small, focused group of people compared to the public. Although book collection in HL does not guarantee an exhaustive list of human differences, willingness of self-disclosure and advocacy likely limit the availability of diverse books and their contents, which would influence the effect of HL. In other words, it is important to investigate organizers’ engagement with the community broadly, and specifically about the approaches in recruitment and selection, including what community relationships are leveraged, any lobbying work completed, possible instances of censorship by organizers or self-stigma by potential books, and any other power imbalance between the organizers and the community that may potentially skew the (re)presentation of human diversity in a HL event.

To answer the aforementioned questions or concerns, more diverse and rigorous research is needed. At the same time, we recognize the challenges in conducting HL research that needs to balance between holding onto a community-based approach and following rigorous research standards and designs. Books are likely to have multiple intersectional identities, which makes conducting sub-group analysis and comparison of specific social groups challenging. HL organized in the real world also does not easily allow experimental manipulation and control. While HLs are intended to engage the community to participate openly and voluntarily, research that asks for participants’ consent may limit who can be recruited and who are able or willing to give such consent, especially when controlled groups are involved. HLs with extensive researcher involvement would risk losing the spontaneity and natural contexts for the free flow of conversation and storytelling. It is important for academic researchers to collaborate with librarians, practitioners, activists, and other community members on the planning, organization, and research of HL initiatives. While the former can contribute to the research designs, ethics, and supports (e.g., funding) that are fundamental to a scientific and rigorous study, the latter groups have the practical wisdom in driving meaningful engagement with different communities and addressing their local needs, altogether of which are conducive to an effective, ethical, and sensible HL. Moreover, future research should employ a typology of research methodologies to create different possibilities of research that provide quality yet diversified evidence for answering different questions about HL. For example, randomized control trials (RCT) can be used to compare HL against other similar interventions or different variations of HL (e.g., in-person, virtual, with technology support) to determine the most effective version. Longitudinal repeated measures can evaluate immediate changes and sustainable effects across time. Intergroup contact theory [37] also provides directions into investigating the mechanism of change, such as the changing roles and identities among readers and books, how a supported norm of acceptance is created in HL, and the differential effects of positive or negative contact experience [35,38]. Drawing from the narrative tradition and storytelling approach [39], conversational and discourse analysis is another useful method to investigate the moment-by-moment interactions and dynamics that illuminate the shift in participants’ perspectives and attitudes. Phenomenological study can focus on understanding the essence and meanings of particular aspects of HL experience, such as narrating traumatic and sensitive stories, motivations and expectations for joining HL, and long-term personal growth in both readers and books.

The current review has several limitations. First, our sample excluded a relatively large number of gray literature compared to a small number of peer-reviewed journal articles and book chapters. Indeed, the gray literature is unrestrained by publication standards and can give more details about insightful practices and challenges. For example, a magazine report of HL events in Beijing gave rich contexts about the considerations to select human books and candidly described examples of failed conversations among participants [40]. Conference proceedings also included great discussions on the conceptual underpinnings of HL [41]. Future research should consider looking into the gray literature to identify HL ideas and facilitate the translation of practical experience into research knowledge. Future reviews can also include non-English search engines and publications to avoid language bias in capturing the global development of HL in diverse linguistic contexts [42]. Furthermore, this review did not evaluate study quality, which might potentially inflate the effectiveness of HL if the studies were not conducted with careful or systematic evaluation methods. Nonetheless, as this study was the first attempt to systematically review the emerging literature of HL, more lenient inclusion criteria allowed us to cover relevant HL experiences without being too restrictive. This current review therefore is descriptive of the state of knowledge and evaluation in this field. Future research using more rigorous evaluation methods and tools would improve the quality of research, thereby allowing the synthesis of more precise intervention effects of HL using, for example, meta-analysis.

## 5. Conclusions

HL is a community-based approach to encourage conversation to promote intergroup contact and understanding among different social groups. A systematic narrative review of 23 academic publications of HL revealed diverse interpretations and implementation in terms of its format, scale, venue, preparation, and recruitment. While the human books were usually the members of minority or marginalized groups, whether the target readers were community insiders or open to the ordinary public depended on the specific goals of a HL event. Both the books and the readers were commonly reported to gain positive experience in their participation, with possible attitudinal change towards the social groups involved. There is a need for a HL guideline that can be flexibly adapted to local customs and cultures, while safeguarding the ethical considerations for all participating parties, especially vulnerable populations. Additionally, the lack of systematic research and clear documentation of implementation methodologies renders the evidence base of HL uncertain. We suggest a more systematic yet naturalistic approach in setting up HLs for future implementation and research.

## Figures and Tables

**Table 1:** Summary of characteristics of included studies.

Authors	Year	Location (City, Region, Country)	Research Methods/Design	Venue	Books Involved (Number; Major or Example Identities)	Readers Involved (Number; Backgrounds)
London and Evans-Lacko [12]	2010	United Kingdom	Descriptive study	N/A	N/A; Individuals who have experience of mental illness	N/A; The public
Clover and Dogus [13]	2014	British Columbia, Canada	Case study	Art gallery	*n* = 10 in research; Artists, organic farmers, burlesque dancers, community police officers, representatives from anti-poverty and sex trade-worker organizations	*n* = 16 in research; The public
Jackson, Huang, and Kasowitz-Scheer [14]	2015	Central New York, United States	Post-survey	Public library and university library	*n* = 15; People suffering from adversity in their lives, such as depression, alcoholism, sexual abuse	*n* = 38; Undergraduates, graduates, staff members, and community members
Orosz, et al. [15]	2016	Hungary	Pre-/Post-survey	N/A	N/A; Roma, LGBT people, homeless	*n* = 105; Public high school students
Sen, McClelland, and Jowett [16]	2016	United Kingdom	Post-survey	University library	*n* = 13 (in 3 HLs); People with lived experience of social work as either service users, carers of users, or practitioners (experience covering mental distress, learning difficulties, physical disabilities, non-verbal communication, childhood trauma, old age, foster care, and terminal illness)	*n* = 204 (in 3 HLs); Social work students joining the program and the course
Gamtso, Mannon, and Whipple [17]	2017	New Hampshire, United States	Post-survey	Public library, high school information center, and university library	Public library: *n* = 12; Gay female rabbi, formerly homeless veteran, Pakistani exchange student, blind social worker, transgender male, and a young woman in substance abuse recovery High school: N/A; N/A University library: N/A; Muslim student from Sudan, transgendered man, recovering alcoholic young adult, Tibetan exile, breast-cancer survivor, antibullying activist, homeschooled student, member of the Deaf community	Public library: N/A; N/A High school: N/A; 11th graders students University library: N/A; Students, faculty, staff, and the general public
Dobreski and Kwasnik [18]	2017	N/A	Qualitative research (content analysis of documents)	N/A	N/A; N/A	N/A; N/A
Blizzard, Becker, and Goebel [19]	2018	Alberta, Canada	Post-survey	University library	N/A; Students, staff, faculty, or community members who are, for example, being transgender, experiencing Islamophobia, recovering from addiction, coping with sexual abuse, surviving breast cancer, being deaf, being autistic	N/A; Students, staff, faculty, or community members
Groyecka, et al. [20]	2019	Wrocław, Poland	Pre-/Post-survey	Public library	N/A; Atheist, person recovered from bulimia and anorexia, ex-prisoner, feminist, freegan, gay, Hindu, HIV+ person, mother of gay, Muslim, German, transgender person, person with schizophrenia, Black person, deaf-blind person, ex-addict, lesbian couple, Roma, Syrian, sober alcoholic, Ukrainian, person on a wheelchair, vegan, Sikh, mother of disabled child, and Jew	*n* = 87 with complete research data; The public
Bagci and Blazhenkova [21]	2020	Turkey	Pre-/Post-survey	University	N/A; Ex-prisoner, HIV+, ex-sex worker, transexual, transexual sex worker, drag queen	*n* = 534 (in 4 HLs); University students
Bordonaro [22]	2020	Southern Ontario, Canada	Post-survey	University library	N/A; Books representing different countries, including Brazil, Cambodia, Canada, China, Ghana, Hong Kong, India, Mexico, Palestine, Serbia, Spain, the United Arab Emirates, the United States of America, and Uzbekistan	*n* = 98; International MBA students (non-native speakers of English) who were assigned for this mandatory activity in their speaking class, and other faculty, staff, and students from the university
Kwan [23]	2020	Hong Kong, China	Qualitative research (practitioner inquiry group discussion)	N/A	*n* = 3 in research; Peer support workers who experienced mental health problems, including schizophrenia and depression	N/A
Schijf, et al. [24]	2020	The Philippines	Pre-/Post-survey	University library	*n* = 84 in actual events, 26 in research (in 9 HLs); LGBTQ, lightweight, obese, teenage mom, human right activist, professional squatter, former drug addict, people with tattoo, tattoo artist, millionaire, young businessman, ex-convict, adopted child, person with HIV, person with bipolar, soldier, police, cancer patient, Muslim	*n* = 1712 in actual events, 973 in research (in 9 HLs); College students, graduate students, teachers, school administrators, and university members from different schools
Yap and Kamilova [25]	2020	Nur-Sultan, Kazakhstan	Post-survey and interview	University library	N/A; People with disabilities (e.g., visual impairment) and LGBTQIA+	N/A; N/A
Halder and Mulliez [26]	2021	Manchester, United Kingdom	Case study	N/A	*n* = 9; Experienced clinicians, including speech and language therapist, dietitian, social worker, occupational therapist, pharmacist, psychiatrist, physiotherapist, psychologist, nurse	*n* = 13; Medical students
Watson [27]	2021	Australia	Post-survey	N/A	N/A; Person with eating disorder, migrant from Germany, transgender woman	N/A; N/A
Chung and Tse [28]	2022	Hong Kong, China	Randomized controlled trial	Student development center	*n* = 2; Woman with schizophrenia and man with bipolar disorder	*n* = 45; Young adults from local tertiary educational institutions who are mentally stable
Giesler [29]	2022	Midwest, United States	Qualitative research (phenomenological case study)	N/A	*n* = 15 in actual event, 11 in research; Veteran with PTSD, person with ASD, person with depression, person with a rare disease, LGBTQ people, victim of sex discrimination, inner-city youth	N/A
Gillum and Williams [30]	2022	Orlando, Florida, United States	Post-survey	Online	*n* = 3 (in a precursor event of HL); Speakers talked about practicing medicine during the HIV/AIDS epidemic in Brazil, implicit bias and inequities in Black, Brown and indigenous peoples, and microaggressions experienced by a young Black female physician	*n* = 19 (in a precursor event of HL); College of Medicine staff and faculty
Jana and Rout [31]	2022	India	Descriptive study	N/A	N/A; N/A	N/A; N/A
van den Dool [32]	2022	The Netherlands (Various cities)	Post-survey	Public library and online	N/A; Homeless, transgender, cross-dressed man, a former member of an Islamic movement in Egypt, a rape victim and a severely disabled woman, adopted children, ex-convicts, asylum seekers, and drag queens	N/A; N/A
Fortune and Leighton [33]	2022	Canada	Qualitative research (interview)	Library	*n* = 10; Individuals living with mental illness, such as bipolar disorder, schizophrenia, anxiety, depression	N/A; Undergraduate students studying therapeutic recreation
Li [34]	2022	Chongqing, China	Case study	University venues (library, reading salon, coffee shop, and outdoors) and online	*n* = 12 (in 12 HLs); Ballet dancer, web celebrity, music student, tea researcher, professors	*n* = 1120 (in 12 HLs; 20 to 200 in each HL); N/A

## Data Availability

No new data were created in this study. Data sharing is not applicable to this article.
